# Inhibition of topoisomerase IIA (Top2α) induces telomeric DNA damage and T cell dysfunction during chronic viral infection

**DOI:** 10.1038/s41419-020-2395-2

**Published:** 2020-03-19

**Authors:** Xindi Dang, Stella C. Ogbu, Juan Zhao, Lam Ngoc Thao Nguyen, Dechao Cao, Lam Nhat Nguyen, Sushant Khanal, Madison Schank, Bal Krishna Chand Thakuri, Xiao Y. Wu, Zheng D. Morrison, Jinyu Zhang, Zhengke Li, Mohamed El Gazzar, Shunbin Ning, Ling Wang, Zhengqiang Wang, Jonathan P. Moorman, Zhi Q. Yao

**Affiliations:** 1 Center of Excellence in Inflammation, Infectious Disease and Immunity, James H. Quillen College of Medicine, East Tennessee State University, Johnson City, TN 37614 USA; 2 Division of Infectious, Inflammatory and Immunologic Diseases, Department of Internal Medicine, Quillen College of Medicine, ETSU, Johnson City, TN 37614 USA; 3 Center for Drug Design, College of Pharmacy, University of Minnesota, Minneapolis, MN USA; 4 Hepatitis (HCV/HBV/HIV) Program, James H. Quillen VA Medical Center, Department of Veterans Affairs, Johnson City, TN 37614 USA

**Keywords:** Cell death and immune response, Hepatitis, HIV infections

## Abstract

T cells play a critical role in controlling viral infection; however, the mechanisms regulating their responses remain incompletely understood. Here, we investigated the role of topoisomerase IIA (Top2α, an enzyme that is essential in resolving entangled DNA strands during replication) in telomeric DNA damage and T cell dysfunction during viral infection. We demonstrated that T cells derived from patients with chronic viral (HBV, HCV, and HIV) infection had lower Top2α protein levels and enzymatic activity, along with an accumulation of the Top2α cleavage complex (Top2cc) in genomic DNA. In addition, T cells from virally infected subjects with lower Top2α levels were vulnerable to Top2α inhibitor-induced cell apoptosis, indicating an important role for Top2α in preventing DNA topological disruption and cell death. Using Top2α inhibitor (ICRF193 or Etoposide)-treated primary T cells as a model, we demonstrated that disrupting the DNA topology promoted DNA damage and T cell apoptosis via Top2cc accumulation that is associated with protein-DNA breaks (PDB) at genomic DNA. Disruption of the DNA topology was likely due to diminished expression of tyrosyl-DNA phosphodiesterase 2 (TDP2), which was inhibited in T cells in vitro by Top2α inhibitor and in vivo by chronic viral infection. These results suggest that immune-evasive viruses (HBV, HCV, and HIV) can disrupt T cell DNA topology as a mechanism of dysregulating host immunity and establishing chronic infection. Thus, restoring the DNA topologic machinery may serve as a novel strategy to protect T cells from unwanted DNA damage and to maintain immune competence.

## Introduction

T cells play a critical role in control of viral infection. In studying the role of T cell dysregulation in viral persistence in humans, we and others have previously shown that chronic viral infections can cause premature T cell aging and immune senescence, as evidenced by the expression of aging markers and particularly, accumulation of DNA damage^1–17^. However, the underlying mechanisms remain unclear.

Given the nature of two intertwined DNA strands in chromosomes, almost all types of DNA activities, including gene replication, transcription, and recombination, can lead to topological entanglements that must be resolved to ensure genetic code normal transactions and cellular functions^18–20^. In order to prevent and correct these topological problems, topoisomerases bind to and cut the DNA strands, allowing the DNA to be untangled, and after that, the DNA backbone is resealed. Failure to complete this catalytic process results in topoisomerase trapping on the DNA termini, forming topoisomerase cleavage complex (TOPcc), and generating protein-linked DNA breaks (PDB), a frequent event that occurs to induce cell apoptosis^21,22^. There are three main types of topology: supercoiling, knotting, and catenation. Correspondingly, the human genome encodes three types of topoisomerases (type IA, type IB, and type IIA) to resolve such DNA entanglements. Notably, the insertion of viral or bacterial DNA into host chromosomes also requires the action of topoisomerases. Many drugs, such as broad-spectrum fluoroquinolone antibiotics and chemotherapy drugs, operate through interference with the topoisomerases of bacteria or cancer cells and create PDB in chromosomal DNA that promote cell apoptosis or dysfunction^23–25^. Thus, although DNA topology is crucial for normal cell functions, its disruption may lead to DNA damage response (DDR) and cell death.

While inhibition of topoisomerases has been widely exploited to kill bacteria and cancer cells^23,24^, the role and mechanisms of topoisomerase in reprogramming DDR and altering the function of T lymphocytes, especially in the context of chronic viral infection, remain largely unknown. We have recently shown that topoisomerase I (Top1) is inhibited and causes topological DNA damage and T cell senescence during chronic viral infections^9^. Here we further demonstrate that Topoisomerase IIA (Top2α) is significantly inhibited and plays a critical role in reprogramming DDR and remodeling T cell function or apoptosis during chronic viral infections.

## Results

### Top2α expression and activity are inhibited in CD4 T cells during chronic viral infections

**Fig. 1:**
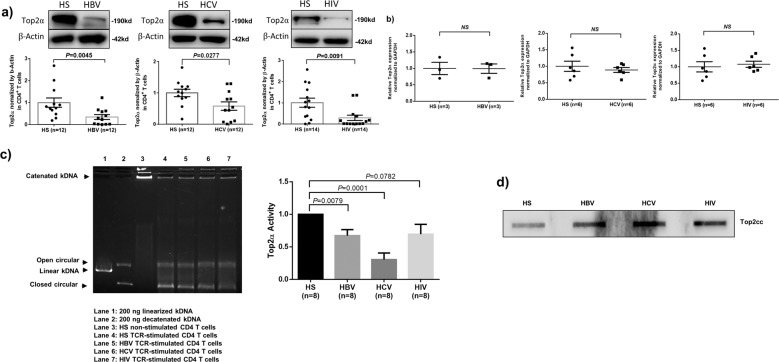
Inhibition of Top2α expression and activity in CD4 T cells during chronic viral infections. **a** Top2α protein expression in CD4 T cells isolated from HBV, HCV, and HIV-infected individuals and healthy subjects (HS). Representative images and summary data of western blot are shown. The Top2α band intensity (lower panel) was normalized to β-actin and is presented relative to HS (*n* = number of subjects). **b** Top2α mRNA levels, determined by RT-qPCR, in CD4 T cells isolated from virally infected individuals and HS. **c** Top2α activity in CD4 T cells isolated from HBV, HCV, and HIV-infected individuals and HS. Representative images and summary data of Top2α-mediated digestion of kDNA substrate (normalized to TCR-stimulated HS control) are shown (*n* = number of subjects tested). **d** Top2cc was detected in genomic DNA isolated from CD4 T cells of virus-infected patients versus HS.

Top2α is critical in unraveling the entangled DNA to prevent unwanted DNA damage and cell death^21^. As an initial approach to explore the role of Top2α in DNA damage and T cell apoptosis, we examined the levels of Top2α in CD4 T cells derived from individuals with chronic viral (HCV, HBV, HIV) infections. Since Top2a is only expressed in activated T cells, we examined Top2a expression in purified CD4 T cells stimulated with anti-CD3/CD28 for 3 days, followed by western blotting. As shown in Fig. 1a, chronically HBV, HCV, or HIV-infected individuals exhibited a lower level of Top2α expression in CD4 T cells compared to age-matched healthy subjects (HS). To determine whether Top2α inhibition occurs at the transcriptional or post-transcriptional level, we measured Top2α mRNA by RT-PCR in CD4 T cells derived from the same subjects. As shown in Fig. 1b, the mRNA levels of Top2α in CD4 T cells isolated from virus-infected patients remained unchanged compared to HS, indicating that Top2α inhibition occurs primarily at the post-transcriptional level during viral infection.

In addition to the Top2α expression, we employed a kDNA-based Top II Assay to measure Top2α activity. We found that the catenated kDNA was extremely large and could not migrate through a 1% agarose gel without being relaxed. In contrast, the monomeric DNA (2.5 kb) rapidly migrated through the gel as nicked open circular or fully closed circular rings. After optimizing concentrations of nuclear extracts, we compared Top2α activity using nuclear extracts from CD4 T cells isolated from virally infected subjects and HS. As shown in Fig. 1c left panel, the linear kDNA (lane 1) and Top2α-relaxed kDNA (lane 2) served as a positive control and the kDNA treated with nuclear extracts of non-stimulated HS T cells served as a negative control (lane 3). The kDNA treated with the optimal concentration of nuclear extracts from T cell receptor (TCR)-stimulated T cells showed varying amounts of decatenated, closed circular DNA, and virally infected patients’ samples (lane 5–7) had lower efficiency in relaxing kDNA compared to HS (lane 4). The relative Top2α activity in CD4 T cells derived from HBV, HCV, and HIV-infected individuals (normalized to HS) was summarized and is shown in Fig. 1c right panel and shows that Top2α activity is inhibited in T cells during chronic viral infection.

To determine if Top2α inhibition results in Top2cc accumulation and entrapment in T cell chromosomes, we measured chromatin-associated Top2cc from CD4 T cells of virally infected patients by immunoblotting with a monoclonal antibody that specifically recognizes covalent Top2α-DNA complexes. As shown in Fig. 1d, an increased amount of Top2cc was detected in the genomic DNA of CD4 T cells isolated from HBV, HCV, and HIV-infected patients compared to HS. These results suggest that T cells from virus-infected patients have DNA topological problems, i.e., Top2α inhibition and Top2cc accumulation.

### Top2α inhibition leads to T cell apoptosis or dysfunction

**Fig. 2:**
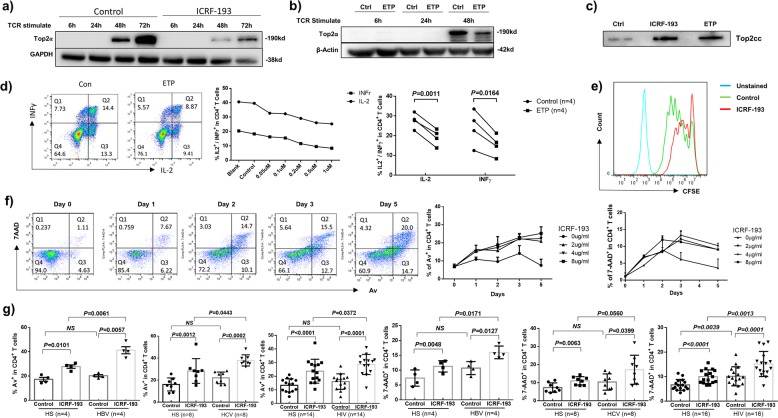
Top2α inhibition induces CD4 T cell apoptosis and dysfunction. **a** Top2α levels in CD4 T cells treated with DMSO (con) or Top2α inhibitor (ICRF-193) in the presence of anti-CD3/CD28 stimulation for 6, 24, 48, and 72 h, were determined by immunoblotting. **b** Top2α protein levels in CD4 T cells that were treated with DMSO (Cont) or Top2α inhibitor (ETP) with TCR (anti-CD3/CD28) stimulation for 6, 24, and 48 h. **c** Top2cc levels in genomic DNA from CD4 T cells that were exposed to DMSO control or Top2α inhibitor (ICRF or ETP) for 72 h, were measured by immunoblotting. **d** Representative dot plots and summary data of IL-2 and IFN-γ expression in TCR-stimulated CD4 T cells that were exposed to ETP or DMSO control. **e** T cell proliferation, measured by CFSE dilution, in TCR-stimulated CD4 T cells in the presence of ICRF and DMSO control for 5 d. **f** Av and 7-AAD staining of CD4 T cells that were treated with various concentrations of ICRF-193 for 1, 2, 3, and 5 days, determined by flow cytometry. **g** The vulnerability of CD4 T cells that were derived from viral infected individuals and HS to the ICRF-193-induced apoptosis, were determined by flow cytometry.

To determine the consequence of Top2α inhibition, we employed two distinct Top2α-targeting compounds in this study: Top2α poison Etoposide (ETP), which binds to and stabilizes Top2cc to prevent DNA religation and generate PDB; and Top2α catalytic inhibitor ICRF-193, which inhibits ATP hydrolysis after strand passage and religation and before the closed clamp conformation reopens^24–29^. Here, we employed Top2α inhibitor-treated primary CD4 T cells as a model to study the role and mechanisms of Top2α in T cell dysregulation. We first assessed the Top2α level in T cells treated with Top2α inhibitors. As shown in Fig. 2a, b, Top2α protein was not detectable in resting primary CD4 T cells but was detected at 48 h after T cell stimulation. TCR-stimulated CD4 T cells showed Top2α inhibition by ICRF or ETP when compared to the DMSO-treated control. Also, Top2α inhibitor-treated CD4 T cells exhibited considerable Top2cc accumulation in their genomic DNA (Fig. 2c). These results indicate that Top2α inhibition and Top2cc accretion in CD4 T cells treated with ICRF and ETP recapitulate our findings in CD4 T cells derived from virus-infected individuals.

We next measured the effect of Top2α inhibition on cellular functions. To this end, CD4 T cells were isolated from HS and exposed to various concentrations (0, 0.05, 0.1, 0.2, 0.5, 1 μM) of ETP in the presence of TCR stimulation for 3 days, followed by measuring intracellular IL-2 and IFN-γ production by flow cytometry. As shown in Fig. 2d, IL-2 and IFN-γ productions were inhibited in a dose-dependent manner in CD4 T cells exposed to ETP. Also, a significant inhibition of T cell proliferation, determined by CFSE dilution in CD4 T cells, was observed in T cells exposed to ICRF compared to control with TCR stimulation (Fig. 2e). In addition, we measured apoptosis of CD4 T cells exposed to various doses (0, 2, 4, or 8 μg/ml) of ICRF for different times (0, 1, 2, 3, or 5 days) in the presence of anti-CD3/CD28 (1 μg/ml) by flow cytometry analysis. As shown in Fig. 2f, ICRF-treated T cells exhibited time-dependent increases in Annexin V (Av) and 7-Aminoactinomycin D (7AAD) staining compared to the DMSO-treated control.

Since T cells from virally infected patients exhibited lower levels of Top2α and enzymatic activity, we hypothesized that these cells are more vulnerable to Top2α inhibitor-mediated cell apoptosis. We thus compared the apoptotic susceptibility of CD4 T cells derived from virus-infected patients and age-matched HS following ICRF treatment. Indeed, CD4 T cells isolated from HBV, HCV, and HIV-infected patients exhibited higher rates of cell apoptosis (Av^+^) and death (7-AAD^+^) upon exposure to a Top2α inhibitor compared to cells from HS (Fig. 2g). Taken together, these results suggest that Top2α inhibition can cause T cell apoptosis or dysfunction, highlighting the role of Top2α in securing T cell survival and function, and providing a robust model to study topological DNA damage in human T cell dysregulation.

### Top2α inhibition induces cell apoptosis by enhancing topological DNA damage that extends to telomeres

**Fig. 3:**
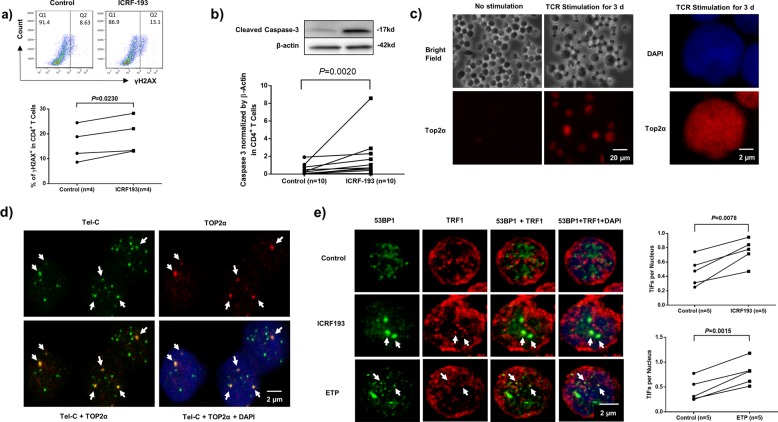
Induction of telomeric DNA damage in CD4 T cells that were treated with Top2α inhibitors. **a** γH2AX levels in TCR-stimulated CD4 T cells treated with ICRF or DMSO control for 3 days, measured by flow cytometry. **b** Active caspase-3 levels in TCR-stimulated CD4 T cells exposed to ICRF or DMSO control for 3 days, measured by immunoblotting. **c** Top2α expression in TCR-activated CD4 T cells, determined by confocal microscopy with white/dark and fluorescent fields. **d** Colocalization of Top2α immunostaining with telomere probe signals in CD4 T cells, determined by confocal microscopy. **e** Representative images and summary data of the dysfunctional telomere-induced foci (TIFs) per nucleus in TCR-stimulated CD4 T cells exposed to ICRF, ETP, or DMSO control for 3 days, determined by confocal microscopy (*n* = number of subjects).

To determine if topological DNA damage is a major cause of T cell apoptosis, we treated primary CD4 T cells with ICRF-193 or DMSO in the presence of TCR stimulation for 3 days, followed by measuring the phosphorylated H2AX (γH2AX), a marker for DNA damage, as well as active caspase-3, a marker for cellular apoptosis, by flow cytometry and immunoblotting. As shown in Fig. 3a, b, increased levels of γH2AX and caspase-3 were observed in ICRF-193-treated CD4 T cells, indicating an increased apoptosis-associated topological DNA damage in T cells with Top2α inhibition.

Since telomeres play an important role in preserving chromosome integrity and cell longevity^30–33^, we asked whether Top2α can also function at telomeres to maintain telomere integrity in proliferating T cells. To test this, we examined Top2α expression in primary CD4 T cells with or without TCR stimulation by microscopy. As shown in Fig. 3c, the expression of Top2α was not detected in resting primary CD4 T cells without TCR stimulation. Similar to the immunoblot results (Fig. 2a), after stimulation with anti-CD3/CD28 for 3 days, Top2α was detected in activated T cells, as shown by positive Top2α immunofluorescent staining in the nuclei of larger, activated T cells upon comparing the same bright-field imaging. Notably, confocal imaging from immunofluorescence-florescence in situ hybridization (IF-FISH) showed that Top2α colocalized with a telomere probe (Tel-C-TAACCC) (Fig. 3d), suggesting a role for Top2α in maintaining telomere integrity.

Human telomeres consist of triple guanine repeats (TTAGGG) that are sensitive to DNA damage^34,35^. We hypothesized that Top2α inhibition-mediated genomic DNA damage may affect telomeres as we have previously shown for telomere erosion in T cells derived from virus-infected subjects^5–9^. To determine telomeric DNA damage in Top2 inhibitor-treated T cells, we measured the number of dysfunctional telomere-induced foci (TIF, a hallmark of telomeric DNA damage^36,37^) by examining the colocalization of 53BP1/TRF1 (p53-binding protein 1/telomeric repeat-binding factor 1) using confocal microscopy. As shown in Fig. 3e, the number of TIFs per nucleus was significantly higher in CD4 T cells exposed to ICRF or ETP compared to vehicle control. These results suggest that Top2α inhibition causes telomeric DNA damage and cell apoptosis.

### Top2α inhibition leads to telomere erosion via suppression of shelterin TRF2 and telomerase activity

**Fig. 4:**
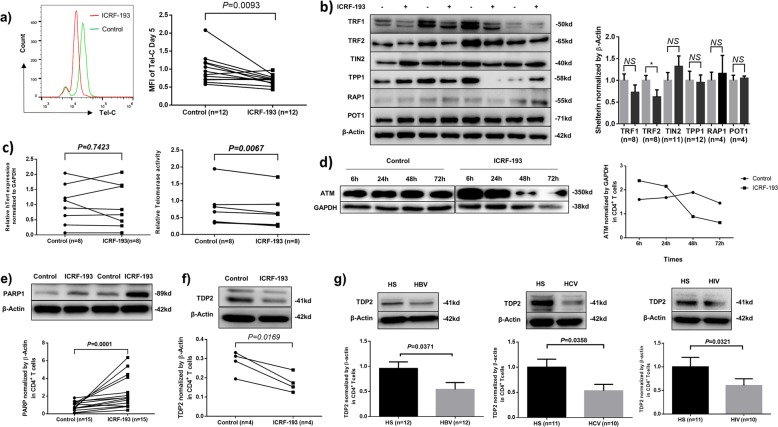
Top2α inhibition induces telomere erosion via suppressing shelterin proteins, telomerase activity, ATM, and TDP2. **a** Telomere length (measured by Flow-FISH) in CD4 T cells treated with ICRF-193 or DMSO control for 5 days. **b** Shelterin proteins (measured by immunoblotting) in CD4 T cells treated with or without ICRF-193. **c** hTERT expression (measured by RT-qPCR) and telomerase activity (measured by TRAP assay) in CD4 T cells treated with or without ICRF. **d** ATM expression (measured by immunoblotting) in TCR-stimulated CD4 T cells exposed to ICRF, or DMSO control for 6, 24, 48, and 72 h. **e** Cleaved PARP1 levels in TCR-activated CD4 T cells exposed to ICRF or DMSO for 72  h. **f** TDP2 expression in TCR-activated CD4 T cells treated with ICRF or DMSO for 72  h. **g** TDP2 expressions in CD4 T cells isolated from HS and chronically HBV-, HCV-, or HIV-infected individuals.

To determine whether Top2α inhibitor-treated T cells mirror the telomere loss seen in patients with viral infections, we measured the telomere length in ICRF-treated T cells by Flow-FISH. T cells treated with ICRF-193 in the presence of TCR stimulation for 5 days showed significantly shorter telomeres compared to the control cells (Fig. 4a).

Telomeres are protected by shelterin proteins^38,39^. To explore the mechanisms of telomere erosion, we examined the integrity of the telomeric shelterin complex in T cells exposed to the Top2α inhibitor ICRF-193. As shown in Fig. 4b, among the shelterin proteins examined, only telomeric repeat-binding factor 2 (TRF2) was significantly inhibited. TRF1 was also slightly downregulated, but TRF1-interacting nuclear protein 2 (TIN2) was slightly upregulated, while telomere protection protein 1 (TPP1), repressor/activator protein 1 (RAP1), and protection of telomere 1 (POT1) remained unchanged. These findings truly recapitulate the results we observed in CD4 T cells derived from HCV and/or HIV-infected patients^6,7^. Since the primary functions of TRF2 are to protect telomeres from unwanted DNA damage and to recruit telomerase to telomeres, its inhibition may lead to telomere uncapping or deprotection as well as telomerase deprivation from telomeres.

Telomeres are replenished by telomerase^32,40,41^. To determine the role of telomerase in Top2α-mediated telomere inhibition, we measured the expression of human telomerase reverse transcriptase (hTERT, the catalytic subunit of telomerase) by RT-PCR and telomerase activities by a TRAP assay in ICRF-193-treated CD4 T cells. As shown in Fig. 4c, ICRF treatment did not change hTERT expression, but remarkably inhibited telomerase activity, which again recapitulated our findings in T cells from patients with HCV or HIV infection.

### Top2α-mediated topological DDR involves dynamic activation and depletion of DNA repair kinases

DNA damage activates the protein kinase ataxia-telangiectasia mutated (ATM), an enzyme involved in repairing double-strand breaks (DSB) for cell survival^42–44^. To determine whether ATM is involved in Top2α-mediated topological DDR, we examined the expression kinetics of ATM in CD4 T cells following ICRF treatments. As shown in Fig. 4d, ATM increased in response to DDR in the early phase (6–24 h) and then gradually diminished with longer ICRF-193 treatments (48–72 h). These results indicate that ICRF-193-induced, Top2α-mediated topological DDR involves dynamic activation and depletion of ATM. These are in line with our recent findings of ATM dynamics in healthy CD4 T cells treated with KML001 or Top1 inhibitor^8,9^ and in CD4 T cells derived from patients with chronic HCV and/or HIV infection^5–7^.

### Top2α-mediated Top2cc accumulation and DDR involves PARP1 induction and TDP2 inhibition

Top2α relaxes intertwined DNA by producing Top2cc, which can be trapped at enzyme-DNA crosslinks and causes PDB, whose removal depends on the tyrosyl-DNA phosphodiesterase-2 (TDP2) pathway^21,22,45^. Notably, Top1cc excision by TDP1 requires Poly ADP-Ribose Polymerase 1 (PARP1), an enzyme that catalyzes the transfer of ADP-ribose onto target proteins and plays an important role in cell apoptosis, DNA repair, and chromosomal stability^46,47^. Specifically, PARP1 binds to and PARylates TDP1, leading to TDP1 stabilization and its recruitment at the sites of Top1cc-PDB to initiate the repair process^47,48^. Whether PARP1 is involved in the TDP2-mediated Top2cc-PDB repair remains unknown. To determine whether Top2α inhibitor-induced DNA damage in T cells involves TDP2 suppression and PARP1 induction, we measured PARP1 and TDP2 in ICRF-treated CD4 T cells. As shown in Fig. 4e, f, the cleaved form of PARP1 was markedly induced while TDP2 was reduced by the treatment with Top2α inhibitor. Notably, TDP2 levels were also significantly inhibited in CD4 T cells derived from HBV, HCV, and HIV-infected subjects (Fig. 4g), which are consistent with the data above showing Top2α inhibition, Top2cc accumulation, PARP1 induction, and apoptosis in these cells (Figs. 1–4)^5–9^.

**Fig. 5:**
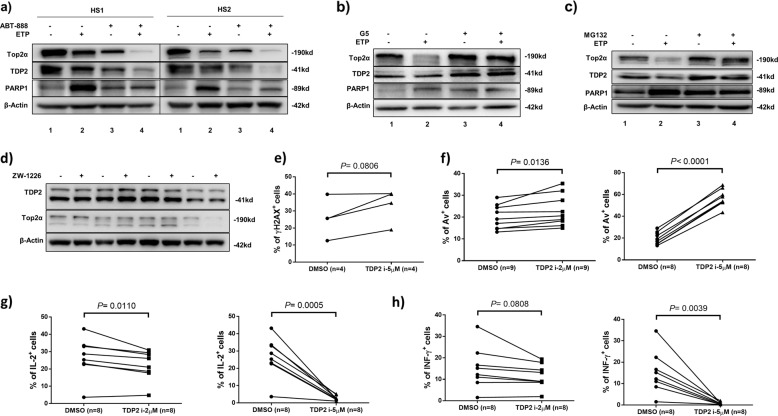
PARP1 and TDP2 are involved in the Top2α-mediated DNA damage and cell apoptosis. **a** Immunoblotting of Top2α, TDP2, and PARP1 expressions in TCR-activated CD4 T cells in the presence or absence of Top2α inhibitor (ETP) or PARP inhibitor (ABT-888). **b** Immunoblotting of Top2α, TDP2, and PARP1 expressions in TCR-stimulated CD4 T cells with or without ETP treatment in the presence or absence of ubiquitin isopeptidase inhibitor (G5). **c** Immunoblotting of Top2α, TDP2, and PARP1 expressions in TCR-activated CD4 T cells with or without ETP treatment in the presence or absence of proteasomal inhibitor (MG132). **d** Immunoblotting of TDP2 and Top2α expressions in TCR-activated CD4 T cells exposed to TDP2 inhibitor (ZW-1226) for 3 days. **e** Flow cytometric analysis of percentages (%) of γH2AX^+^ cells within TCR-activated CD4 T cells treated with TDP2 inhibitor (5 μM) or DMSO control for 3 days. **f**–**h** Flow cytometric analysis of percentage (%) of Av^+^, IL-2^+^, and IFN-γ^+^ cells within TCR-activated CD4 T cells treated with TDP2 inhibitor (2 μM, 5 μM) or DMSO control for 3 days.

We next assessed whether inhibition of PARP1 could increase DNA damage in ETP-treated T cells. As shown in Fig. 5a, compared to the DMSO-treated control (lane 1), ETP treatment (lane 2) inhibited Top2α and TDP2 expressions, but increased the cleaved form of PARP1. Notably, the PARP1 inhibitor (ABT-888)^49^ diminished Top2α and TDP2 expressions, but increased PARP1 cleavage in DMSO-treated cells (lane 3) as well as in ETP-treated cells (lane 4), suggesting that PARP1 is involved in the ETP-mediated Top2α inhibition and TDP2-mediated DNA repair pathway.

We further hypothesized that Top2cc-mediated transcription blockade may trigger Top2α protein degradation by an ubiquitin-mediated mechanism. To test this hypothesis, we examined whether the ubiquitin degradation machinery could contribute to the observed Top2α protein inhibition and DNA damage-mediated apoptosis. As shown in Fig. 5b, compared to the control (lane 1), ETP treatment decreased Top2α and TDP2 but increased the PARP1 levels (lane 2). Intriguingly, inhibition of ubiquitin by the isopeptidase inhibitor (G5), which causes depletion of free nuclear ubiquitin^50^, prevented Top2α and TDP2 degradation in DMSO-treated cells (lane 3), especially in ETP-treated T cells (lane 4). We also investigated whether inhibition of the ubiquitin system could prevent DDR-mediated cell apoptosis. Treatment with G5 prevented further induction of PARP1 in ETP-exposed cells (lane 4) compared to DMSO-treated control (line 3) and ETP-treated cells (lane 2), although G5 treatment alone (lane 3) increased level of cleaved PARP1 compared to cells exposed to DMSO (lane 1). Similarly, inhibition of proteolysis by the proteasome inhibitor MG132, which prevented ETP-induced Top2α and TDP2 degradation, also prevented ETP-induced accumulation of PARP1 (Fig. 5c), suggesting that topological DNA damage depends on Top2α and TDP2 ubiquitination and proteasome degradation. Taken together, these results suggest that the ubiquitin-mediated proteolysis of Top2α and TDP2 is involved in the ETP-induced topological DNA damage.

### TDP2 inhibition promotes CD4 T cell apoptosis and dysfunction

Since Top2α-mediated topological DNA damage can be repaired by TDP2, which is also inhibited in CD4 T cells treated with Top2α inhibitor in vitro and isolated from virally infected patients in vivo (Fig. 4), we next determined the effect of the TDP2 inhibitor ZW-1226 on T cell survival and functions. ZW-1266 is a cell-permeable deazaflavin analog that selectively inhibits TDP2 activity and sensitizes cancer cells toward ETP-treatment^51^. While this drug did not affect the expression levels of TDP2 and Top2α in TCR-activated CD4 T cells (Fig. 5d), TDP2 inhibition resulted in an increased level of γH2AX and Av staining, and decreased intracellular IL-2 and IFN-γ production (Fig. 5e–h), suggesting that TDP2 is important for maintaining Top2α-mediated T cell DNA topology and cellular functions.

## Discussion

T cells play a pivotal role in controlling pathogenic infection and vaccine responses. During chronic viral infections, however, T cells are always dysregulated and often non-responsive to vaccines^1^. We and others have previously reported that T cells from chronically virus-infected individuals are prematurely aged due to accelerated telomere erosion^1–17^, but the underlying mechanisms for T cell telomeric DNA damage remain unclear. Since Top2α is required to remove DNA supercoiling generated during cell proliferation, and Top2cc can become trapped during gene transcription to cause Top2cc-linked PDB due to TDP2 depletion^21,22^, we hypothesized that DNA topology in T cells may be affected during viral infections to trigger DDR as a mechanism of virus-induced immune evasion, and thus, persistent infection.

In the present study, we employed T cells isolated from virus-infected individuals and primary T cells treated with Top2α inhibitors as model to obtain molecular insights into the mechanisms underlying Top2α-mediated DNA damage and repair signaling. We demonstrated that: (1) T cells derived from chronically virus-infected individuals exhibit diminished Top2α enzyme expression and activity, leading to accumulation of Top2cc and DNA damage, including telomere erosion; (2) ETP or ICRF-induced Top2α inhibition, topological DNA damage, telomere uncapping and attrition, T cell apoptosis and dysfunctions recapitulate the phenotype seen in T cells during chronic viral (HBV, HCV, HIV) infections, highlighting the role of Top2α in maintaining telomeric DNA integrity and securing T cell survival or function; (3) Top2α inhibition occurs at the post-transcriptional level (likely via the ubiquitin-mediated proteolysis) and is related to TDP2 suppression, PARP1 induction, and Top2cc accumulation; (4) Top2α inhibition-mediated telomeric DDR involves telomere TRF2-uncapping, diminished telomerase activity, and a dynamic ATM activation followed by deprivation; and (5) T cells from virally infected subjects with lower Top2α levels are more vulnerable to Top2α inhibitor-induced topological DNA damage and cell apoptosis, indicating an important role for Top2α in preventing unwanted DNA damage and securing cell survival.

**Fig. 6:**
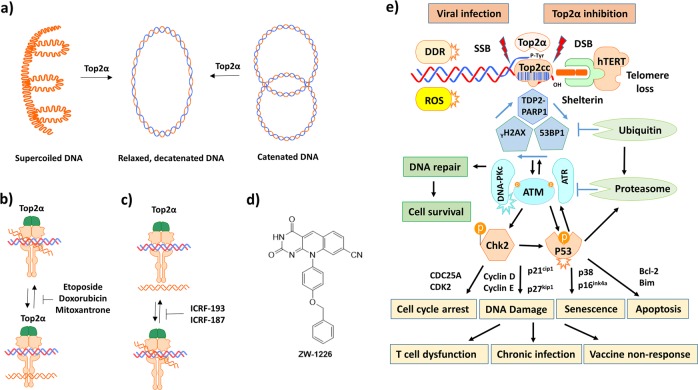
A working model for virus-induced disruption of DNA topology and in T cell dysregulation. **a** A schematic representation of Top2α on the supercoiled, catenated chromosomal DNA. Top2α cuts both strands of the DNA helix in order to manage DNA tangles and supercoils. Once cut, the ends of the DNA are separated, a second DNA duplex is passed through the break, and the cut DNA is then religated. This process allows Top2α to increase or decrease the linking number of a DNA loop by 2 units, and thus promotes chromosome disentanglement. **b**, **c** A schematic representation showing ETP or ICRF in the Top2α-DNA crosslinks. Etoposide is a semisynthetic derivative of podophyllotoxin that forms a ternary complex with DNA and Top2α, and prevents religation of the DNA strands, causing DNA strand breaks. ICRF-193 poisons DNA-Top2α crosslinking complexes and delays cell cycle progression to mitosis. **d** Structure of ZW-1226, a TDP2 functional inhibitor. **e** A schematic model of Top2α-mediated telomeric DDR and T cell dysregulation during chronic viral infection. The intertwined nature of two complementary DNA strands often leads to topological entanglements during DNA replication, transcription, and recombination that must be resolved to ensure normal DNA transactions and cell functions. In order to prevent and correct these types of topological problems, Top2α binds to DNA and cut two DNA strands simultaneously, allowing the DNA to be untangled or unwound. Based on our findings, the immunomodulatory virus (HBV, HCV, HIV) infection and/or ROS-generating inflammation can inhibit Top2α protein expression and enzyme activity, leading to Top2cc becoming trapped at the DNA break sites including telomere termini, and causing topological DNA damage, telomere loss, cell senescence, and apoptosis. This continuous regulatory cascade represents a novel molecular mechanism underlying CD4 T cell dysfunction, which contributes to the viral persistence and vaccine non-responsiveness in human viral infections.

Although the accumulation of DNA damage and the failure to repair it may affect cell survival and function, the molecular signaling pathways in T lymphocytes in the context of chronic viral infections are incompletely understood. Top2α cuts both strands of the DNA helix simultaneously in order to manage DNA entangles and supercoils^25^. Once cut, the ends of the DNA are separated, a second DNA duplex is passed through the break, and the cut DNA is then religated. This process allows the Top2α to increase or decrease the linking numbers of a DNA loop by two units and promotes chromosome untying (Fig. 6a). Etoposide is a semisynthetic derivative of podophyllotoxin that forms a ternary complex with DNA and Top2α and prevents religation of the DNA strands, and by doing so causes the DNA strands to break (Fig. 6b)^26^. ICRF-193 is a bisdioxopiperazine Top2α catalytic inhibitor that blocks Top2α turnover by trapping it in a closed clamp conformation and delays the cell cycle progression from metaphase in mammalian cells (Fig. 6c), likely with the ability to damage DNA and trap the DNA-Top2α crosslinking complex^27^. ZW-1266 is a cell-permeable deazaflavin analog (Fig. 6d) that selectively inhibits TDP2, but not TDP1 enzymatic activity in vitro, and strongly sensitizes cancer cells toward the treatment of ETP, a phenotype consistent with the TDP2 function loss and leads to the Top2cc formation^51^. Cancer cells and highly proliferative T cells rely on Top2α more than other cells, because they divide more rapidly. Therefore, deficiency or inhibition of this enzyme causes errors in DNA topology and promotes cell apoptosis. Our results, in conjunction with our previous reports^5–9^, support a model (depicted in Fig. 6e), in which Top2α inhibition and Top2cc accumulation block transcription elongation, which triggers the ubiquitin-mediated Top2α proteolysis and the generation of Top2cc-mediated PDBs. Defective repair of these PDBs by the DNA repair machineries such as TDP2 can lead to more DDR, which activates ATM kinase and phosphorylation of its downstream substrates such as CHK2 and p53. Activated ATM also activates 53BP1 and γH2AX assembly into nuclear DNA damage foci and promotes the ubiquitination of multiple signaling molecules in the process of DNA damage and repair. Notably, several E3 ligases have been reported for Top2α ubiquitination and proteasomal degradation^45^. Our findings that Top2α and TDP2 inhibitions, as well as γH2AX and PARP1 inductions in treated T cells, depend on Top2α ubiquitination suggest that they arise during the repair of Top2cc, and this pathway feeds back to enhance Top2cc repair after Top2α-linked PDB induction. Moreover, it appears that PARP1 is involved not only in the process of TDP1-mediated excision of Top1cc^47,48^, but also in TDP2-mediated repair of Top2cc, since PARP1 inhibition alters Top2α-PDB induction following Top2cc stabilization. This Top2 model, similar to our Top1 model^9^, is supported by our recent findings showing that ATM deficiency^5,7^, TRF2 uncapping^6^, and telomere targeting^8^ promote telomeric DNA damage and T cell senescence and apoptosis, as demonstrated in this study in T cells isolated from chronic viral infections or T cells treated with Top2α inhibitors.

While the mechanisms leading to Top2α inhibition during chronic viral infections remain unclear, multiple factors may play a role. Topological DNA damage might occur in proliferating T cells under physiological conditions, but Top2cc can be trapped under a broad range of pathological conditions, including Top2α inhibition by immuno-modulating viruses (HBV, HCV, and HIV), oxidative base damage by alkylation with carcinogenic compounds or antiviral agents, and ribonucleotide misincorporations during genetic activities in over-expanding T cells in response to low-grade chronic inflammation^18–23^. Therefore, these Top2α-linked PDBs may have a significant impact on replicative T cells, leading to reprogramming of the DDR and remodeling of T cell fate that arise from the defective removal of Top2cc due to deprivation of TDP2, deprotection of telomeres by shelterins^6^, failure of telomere elongation by telomerase, and deficiency of DNA damage repair by ATM^5,7^. T cells may particularly be prone and vulnerable to Top2α-mediated topological DNA damage and cell apoptosis as a result of high rates of cell turnover and oxygen consumption, which produce large amounts of reactive oxygen species (ROS) that we have recently shown significantly elevated in T cells from viral (HCV and HIV) infected individuals^5–7^. Indeed, ROS can stabilize Top2cc to cause topological DNA damage and activate the ATM signaling pathway^52^. Thus, our findings suggest a new concept in which topological DNA damage contributes to T cell senescence, apoptosis, and immune evasion during chronic viral infections.

It should be pointed out that while Top2α inhibition and Top2cc accumulation explain both telomeric DNA damage and cell apoptosis, they may also function as a double-edged sword that can result in both overwhelming cell death storm in acute infection and immune tolerance or immune suppression in chronic infection^1^. Nevertheless, these novel findings demonstrate, for the first time, the role of Top2α in DDR and shed light on the molecular aspects of immunomodulation during human viral infections. Most importantly, this study provides a potential strategy for restoring impaired DNA topological machinery as a mean to improve T cell functions and vaccine responses against human viral diseases.

## Materials and methods

### Subjects

The study protocol was approved by the institutional review board (IRB) of East Tennessee State University and James H. Quillen VA Medical Center (ETSU/VA IRB, Johnson City, TN). Written informed consent was obtained from all subjects. The study subjects were composed of four populations: 27 chronically HBV-infected patients on antiviral treatment with undetectable viremia (HBV-DNA); 42 chronically HCV-infected patients prior to antiviral therapy; 56 latently HIV-infected patients on antiretroviral therapy (ART) with undetectable viremia (HIV-RNA); and 105 age-matched healthy subjects (HS). HS blood samples were provided by Physicians Plasma Alliance (PPA), Gray, TN, and were negative for HBV, HCV, and HIV infections. The characteristics of the subjects recruited in this study are described in Table 1.

**Table 1:** Demographic information of the study participants.

Subjects	Numbers	Age (Mean)	Gender (M/F)	Viral load and other characteristics
HCV	42	29–68 (48)	36/6	135–6,696,048 IU/ml, 32 GT1, 6 GT2, 4 GT3
HBV	27	29–72 (42)	22/5	All on antivirals with undetectable HBV-DNA
HIV	56	22–70 (48)	46/10	All on ART with undetectable HIV-RNA
HS	105	21–65 (46)	75/30	All tested negative for HCV, HBV, and HIV

### Cell isolation and culture

Peripheral blood mononuclear cells (PBMCs) were isolated from whole blood by Ficoll density centrifugation (GE Healthcare, Piscataway, NJ). The CD4^+^ T cells were isolated from PBMCs using the CD4^+^ T Cell Negative Selection Kit and a MidiMACS™ Separator (Miltenyi Biotec Inc., Auburn, CA). The cells were cultured in RPMI-1640 medium supplemented with 10% FBS (Atlanta Biologicals, Flowery Branch, GA), 100 IU/ml penicillin, and 2 mM L-glutamine (Thermo Scientific, Logan, Utah) and maintained at 37 °C and 5% CO_2_ atmosphere.

### Flow cytometry

Intracellular IL-2, IFN-γ cytokine production, DNA damage marker γH2AX expression, CSFE-label CD4 T cell proliferation, telomere length measured by Flow-FISH, and cell apoptosis assay for Av/7AAD expression were analyzed by flow cytometry, as described previously^5–7^. The following reagents were used in the assays: PE-labelled Av, 7-AAD, IL-2, IFN-γ, γH2AX (BD), CD4-Alexa-647 (Bio legend), telomere probe Tel-C (TAACCC)-FITC (0.25 µg probe/mL, PNA Bio, Newbury Park, CA).

### RNA isolation and real-time RT-PCR

Total RNA was extracted from 1 × 10^6^ CD4 T cells using the PureLink RNA Mini Kit (Invitrogen, Carlsbad, CA), and cDNA was synthesized using the High Capacity cDNA Reverse Transcription Kit (Applied Bio-systems, Foster City, CA) per the manufacturer’s instruction. Quantitative RT-PCR was performed in triplicate as described previously^5–7^. Gene expression levels were determined using the 2^-ΔΔct^ method. Values were normalized to the GAPDH level and are presented as fold changes. The PCR primer sequences are shown in Table 2.

**Table 2:** Primer sequences used in real-time RT-PCR of this study.

Primers	Forward	Reverse
hTERT	5′-CCAAGTTCCTGCACTGGCTGA-3′	5′-TTCCCGATGCTGCCTGACC-3′
Top2α	5′-ACCATTGCAGCCTGTAAATGA-3’	5′-GGGCGGAGCAAAATATGTTCC-3’
GAPDH	5′-TGCACCACCAACTGCTTAGC-3′	5′-GGCATGGACTGTGGTCATGAG-3′

### Immunoblotting

Since Top2α is only expressed in activated T cells, we examined Top2α level in purified CD4 T cells activated with anti-CD3/CD28 for 3 days by immunoblotting. Briefly, CD4 T cells (2 × 10^6^) were purified from HCV, HBV, or HIV patients and HS as described previously^9^. Primary and secondary antibodies included Top2α, PARP1, TRF1, TRF2, TPP1, TIN2, RAP1, POT1, ATM, GAPDH, β-actin, and horseradish peroxide-conjugated antibody (Cell Signaling). Images were captured using ChemiDoc™ XRS + System (Bio-Rad). Protein band intensity was quantitated by the Image Lab software (Bio-Rad).

### Top2α activity assay

The activity of Top2α was measured using the Topoisomerase II Assay Kit (Cat #TG1001; Topogen Inc; Buena Vista, CO). Briefly, CD4 T cells were isolated, and cell extracts were prepared according to the manufacturer’s instructions. Because nuclease activity may cause some degradation of the kDNA substrate and generate a smear of degradation products, we used a Top2α isolation kit to purify the nuclear extract. The purified extract was mixed with plasmid DNA substrate and reaction buffer for 30 min at 37 °C, loaded to a 1% agarose gel with loading dye and then subjected to electrophoresis for 2 h at 5–10 V/cm before illuminating with a UV transilluminator. The intensity of the relaxed circular DNA was measured by densitometry.

### Top2cc detection

Top2cc was detected using the Human Topoisomerase ICE Assay Kit (Cat #TG1020-2a; Topogen). The method of DNA purification was modified by combining the ICE Assay Kit and PureLink™ Genomic DNA Mini Kit (Cat #K182001; Thermo Fisher Scientific, Waltham, MA). Briefly, genomic DNA samples were extracted from cell pellets using buffers from the ICE assay kit and then purified by the column of PureLink™ Genomic DNA Mini Kit. The DNA samples were loaded onto NC membrane by a vacuum pump and were incubated with primary anti-Top2cc antibody from the ICE assay kit, followed by western blotting as described above.

### Confocal microscopy

CD4 T cells were isolated and cultured as described above, followed by immunofluorescence staining using a method described previously^6^. The primary antibodies included Rabbit anti-53BP1, and mouse TRF1 (Thermo Fisher). The secondary antibodies included anti-rabbit IgG-Alexa Fluor 488 and anti-mouse IgG-Alexa Fluor 555 (Invitrogen). We also used Tel-C (TAACCC)-FITC probe for telomere staining. The cells were washed and mounted with DAPI Fluoromount-G (SouthernBiotech, Birmingham, AL). Images were acquired with a confocal laser-scanning inverted microscope (Leica Confocal, Model TCS sp8, Germany).

### Statistics

The data were analyzed using Prism 7 software and are presented as mean ± SEM. Differences between two groups were analyzed by independent Student’s *t*-test or paired *t*-test. *P*-values of <0.05, or <0.01 were considered statistically significant or very significant, respectively.
